# Break-through amplified spontaneous emission with ultra-low threshold in perovskite via synergetic moisture and BHT dual strategies

**DOI:** 10.1038/s41377-025-02171-8

**Published:** 2026-02-02

**Authors:** Dingke Zhang, Rui Li, Haoyue Luo, Zhen Meng, Jingwen Yao, Hongfang Liu, Yexiong Huang, Shuaiqi Li, Peng Yu, Jie Yang, Mingyu Pi, Shencheng Fu, Zhenxiang Cheng, Yichun Liu

**Affiliations:** 1 School of Physics and Optoelectronic Engineering, Chongqing Normal University, Chongqing, China; 2 School of Physics, Northeast Normal University, Changchun, China; 3 State Key Laboratory of Integrated Optoelectronics, Northeast Normal University, Changchun, China; 4 Institute for Superconducting and Electronic Materials, Faculty of Engineering and Information Sciences, University of Wollongong Innovation Campus, North Wollongong, NSW Australia

**Keywords:** Semiconductor lasers, Laser material processing

## Abstract

Micro-nano lasers hold significant promise for on-chip integrated photonics, where perovskite materials emerge as compelling gain media despite stability challenges. While moisture typically degrades perovskite structures, its controlled integration can paradoxically enhance crystallization. Here, we demonstrate a synergistic strategy utilizing water molecules and butylated hydroxytoluene (BHT) additive to achieve high-quality methylammonium lead iodide (MAPbI₃) films with low defect density. Through optimized BHT (4 wt%) combined with 95% relative humidity treatment, we attain an unprecedented amplified spontaneous emission (ASE) threshold of 8.987 μJ cm⁻² under nanosecond pulse excitation – the lowest value reported to date. This dual-triggered film completes ASE intensity retention after 30-day ambient storage. In situ structural and optoelectronic characterization reveals that BHT extends water-perovskite interaction, facilitating organic cation vertical diffusion and preferential (110)-oriented crystallization with 53.02% perpendicular alignment. Transient absorption (TA) spectroscopy confirms suppressed non-radiative recombination, evidenced by 11% prolonged carrier lifetime (6145 ps), while temperature-dependent photoluminescence reveals enhanced exciton binding energy (73.50 meV vs. 60.68 meV) conducive to low-threshold lasing. This work transforms moisture from a degradation agent into a crystallization promoter, establishing a paradigm for high-performance perovskite lasers with simultaneous efficiency and stability.

## Abstract (web summary)

Break-through ASE with ultra-low threshold in Perovskite based on a synergistic dual-regulation strategy leveraging water molecules and additive of BHT.

## Graphical abstract

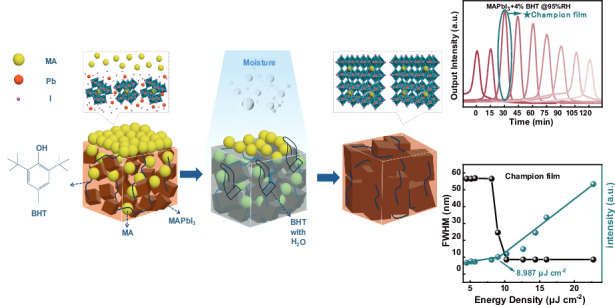

## Introduction

Micro-nano lasers offer compelling advantages for integrated photonic systems, including micrometer-scale footprint, high beam output quality, narrow emission linewidth and low power consumption^1,2^. Perovskite materials have emerged as exceptional gain media in this field due to their superior optoelectronic properties, tunable bandgaps, and high optical gain coefficients^3–6^. Their balanced ambipolar charge transport further suggests strong potential for electrically pumped lasers^6–10^. However, the susceptibility of perovskite films to environmental degradation, particularly through moisture ingress along grain boundaries, poses significant challenges for the application in the field of lasers. Exposure to humid environments accelerates the decomposition of organic-inorganic hybrid perovskites into lead iodide (PbI_2_), severely compromising device performance^11,12^. This instability necessitates urgent solutions for enhancing both crystallization quality and environmental robustness.

Intriguingly, while moisture negatively influences perovskite stability, a suitable amount of water molecules can also activate the crystallization process of perovskite. Water molecules reversibly adsorbed on film surfaces facilitate ion migration and recrystallization, while creating aqueous microenvironments that extend precursor diffusion paths to promote grain growth^13–16^. Apparently, water molecules mimic a double-edged sword for perovskite-based devices, which can affect the orientation, growth, structural integrity and crystallization of perovskite. Excess water molecules impair perovskite crystallinity and accelerate perovskite degradation through PbI_2_ precipitation_._ On the other side, a moderate water content facilitates the crystal formation and enables a spatially homogeneous intermediate phase, resulting in high-quality perovskites with significantly suppressed defects. This dual nature manifests in humidity-dependent crystallization outcomes: 40% relative humidity (RH) annealing enhances texture orientation^17^, whereas 50% RH induces undesirable non-perovskite phases^18^. The narrow window for the beneficial crystallization process, coupled with persistent defect formation (e.g., under-coordinated Pb^2+^ and I^−^ ions at grain boundaries or surface)^14,19^ that promotes unexpected non-radiative recombination^13,20,21^, underscores the need for precise moisture regulation strategies.

Additive engineering presents a promising approach to address these challenges. Molecular additives can simultaneously optimize morphology, crystallinity, passivate defects, and enhance stability^22–27^. Butylated hydroxytoluene (BHT), an antioxidant with low phenolic hydrogen bond dissociation energy (BDE), improves device performance by modulating precursor chemistry and suppressing the intermediate decay pathways^28,29^. However, existing strategies relying solely on additive control fail to fully exploit moisture’s beneficial effects on perovskite film quality, which cannot further unleash their excellent photoelectric application potential.

Herein, we propose a synergistic dual-regulation strategy leveraging both water molecules and additive of BHT for achieving the quality improvement of perovskites. Capitalizing on BHT’s hydrophobic antioxidant properties, we prolong beneficial water-perovskite interactions, improving its morphology, crystallization and stability. Moisture and BHT dual triggered methylammonium lead iodide (MAPbI_3_) perovskite film is achieved with an ultralow amplified spontaneous emission (ASE) threshold of 8.987 μJ cm^−2^ (lowest reported under ns-pulse excitation). In situ structural evolution monitored via XRD/GIWAXS/SEM reveals BHT-mediated moisture control enables vertical organic cation diffusion and preferential (110) orientation (53.02% perpendicular alignment). The ASE properties are explored via excitation dynamics and population inversion analysis. Transient absorption (TA) spectroscopy confirms suppressed non-radiative recombination, while 1H NMR and X-ray photoelectron spectroscopy (XPS) elucidate hydration kinetics. This work establishes a paradigm for harnessing environmental factors to achieve high-performance perovskite lasers with unprecedented efficiency-stability balance.

## Results

### Effect of BHT on improving the perovskite film quality and ASE

**Fig. 1:**
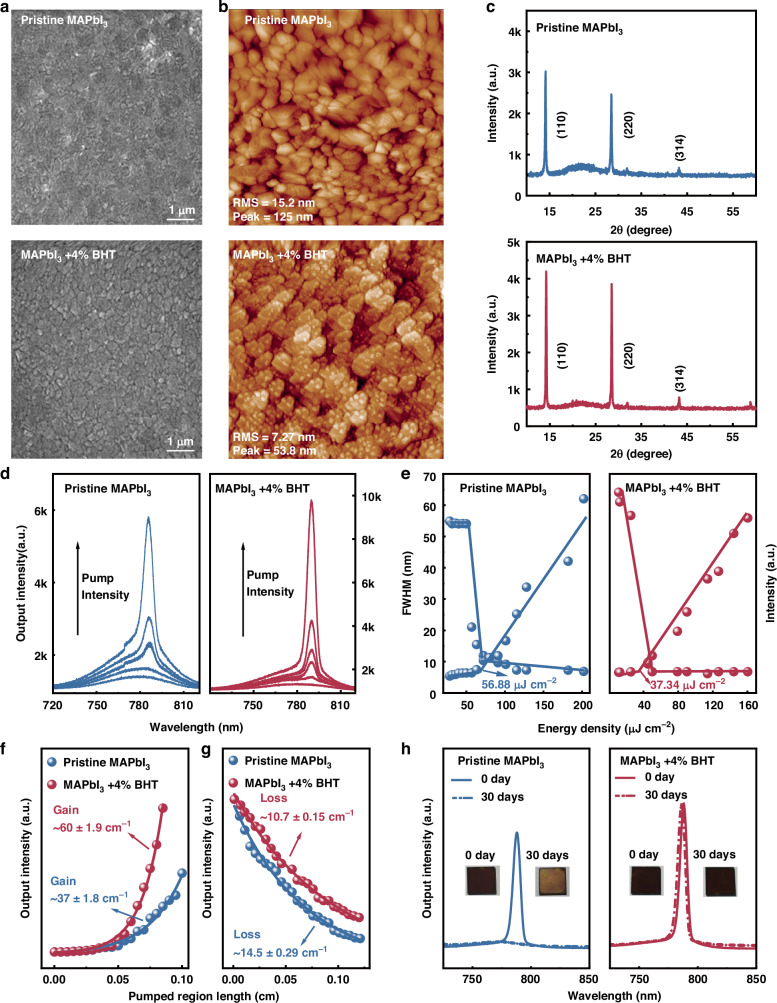
Morphological and optical characterizations. **a** SEM, **b** AFM, **c** XRD patterns, **d** emission spectra, **e** ASE threshold, **f** gain, **g** loss and **h** long-term stability of the pristine MAPbI3 and MAPbI3+4% BHT films. Insets in **h** are photographs of both films after 30 d storage under ambient conditions

BHT, as an additive added to the precursor solution in perovskite film preparation, was used to reduce the film defects, improve their morphology and crystallinity, and passivate the defect states. In this study, the exclusive effect of BHT on the film quality and ASE was investigated by adding different concentrations of BHT (0, 2, 4, 6 and 8 wt.%) to MAPbI_3_. The optimized BHT concentration was selected for fabricating a high-quality film. Figures 1a and S1a present the SEM images of MAPbI_3_ films with varying BHT concentrations, which reveals that the grain size of the as-prepared perovskite films alters on changing BHT concentrations. The sample with 4% BHT exhibits highly compact grains with an average maximum size. Large grains reduce the grain boundary density inducing few defects, which facilitates the transport and accumulation of photogenerated charges. It should be mentioned that excess BHT can disrupt the grain formation due to its high viscosity resulting in an uneven distribution on the film surface (Fig. S2). The atomic force microscopy (AFM) results depicted in Figs. 1b and S1b show a minimum surface roughness (7.27 nm) for the sample of MAPbI_3_+4% BHT, indicating that the optimized BHT concentration (4%) effectively improves film smoothness. The crystallinity of MAPbI_3_ perovskite films in the presence of different BHT concentrations was analyzed via XRD, as shown in Figs. 1c and S1c. The peaks at 14.1°, 28.5° and 43.2° represent the (110), (220) and (314) planes of the tetragonal structure of perovskite, respectively. The absence of additional diffraction peaks suggests that the tetragonal crystallinity of perovskite grains is improved by BHT. The MAPbI_3_+4% BHT film exhibited an optimal film quality: large compact grains, smooth surface and high crystallinity. These characteristics can be attributed to the π–π bonding of the benzene rings accumulated near grain boundaries and the cross-linking of the adjacent grains through hydrogen bonds, promoting perovskite grains to grow and form large grain sizes for increasing the surface coverage^30,31^.

Perovskite materials with high gain characteristics have been proven to have great potential in on-chip light sources. The effect of BHT on the gain characteristics of the MAPbI_3_ film was further studied. The ASE performance of MAPbI_3_ films with different BHT concentrations was assessed using a 355 nm nanosecond pulsed laser (5 mm long and 0.8 mm wide pump band) for exciting the as-prepared perovskite films under ambient conditions. This setup was chosen to excite the MAPbI_3_ perovskite films using a simple pump source at a low energy level. Figure 1d shows the emission spectra at different pump energy fluxes of pristine MAPbI_3_ and MAPbI_3_+4% BHT films. The output emission intensity and full width at half maximum as a function of the pump intensity are used to evaluate the ASE threshold value, as shown in Fig. 1e (The output emission spectra and ASE threshold values of the samples with different BHT concentrations are shown in Fig. S3), which indicates a distinct knee mark as the ASE threshold of the films in the graph. Notably, the ASE thresholds of all BHT-added MAPbI_3_ films are lower than that of the pristine MAPbI_3_ film (56.88 μJ cm^−2^), and the MAPbI_3_+4% BHT film exhibits the lowest threshold value (37.34 μJ cm^−2^). The ASE performance of pristine MAPbI_3_ and BHT-added MAPbI_3_ films is further analyzed by evaluating their net gain and loss (Figs. 1f, g, and S4), BHT-added MAPbI_3_ film exhibit higher net modal gain and lower loss factor than those of the pristine MAPbI_3_ film based on Equations. S1 and S2 (Supplemental Material), and the MAPbI_3_+4% BHT film shows the best performance (Table S1). Thus, the addition of optimized 4% BHT during perovskite film preparation improves the film quality and reduces the defect density, leading to the reduced nonradiative recombination in the perovskite film.

Space charge limited current was employed to evaluate the defect state within the pristine MAPbI_3_ and MAPbI_3_+4% BHT perovskite films. The trap-state density *N*_*t*_ can be obtained by the formula: *V*_*TFL*_=$\frac{q{N}_{t}{L}^{2}}{2\varepsilon {\varepsilon }_{0}}$, where *L* represents the thickness of the perovskite films, *ε*_*0*_ denotes the vacuum permittivity (*ε*_*0*_ = 8.85 × 10^−12^ F m^−1^), *q* stands for the elementary charge and *ε* refers to the dielectric constants of the perovskite which are typically ≈35^32^. For a given thickness of the samples, the total *N*_*t*_ is directly proportional to the trap-filled limit voltage (*V*_*TFL*_). As shown in Fig. S5, the pristine MAPbI_3_ film displays a *V*_*TFL*_ of 1.46 V while the MAPbI_3_+4% BHT perovskite film displays a *V*_*TFL*_ of 0.71 V, showing the total trap density decreased from 2.73 × 10^17 ^cm^−3^ in the control film to a lower value of 1.26 × 10^17 ^cm^−3^ in the MAPbI_3_+4% BHT film. These results confirm that BHT treatment is advantageous for obtaining a high-quality perovskite film with a much-reduced defect state.

Besides improving the film quality, BHT was selected as an additive for perovskite films owing to its antioxidant properties, which can efficiently improve the stability of perovskites under ambient conditions. The stability evaluation of pristine MAPbI_3_ and MAPbI_3_+4% BHT films are presented in Fig. 1h. The ASE output intensities of the pristine MAPbI_3_ film decrease remarkably from 100% to 5.8% after 30 d storage under ambient conditions. By contrast, about 100% of ASE output intensity for the MAPbI_3_+4% BHT film is retained after the same storage period. The photographs in the inset of Fig. 1h indicate a noticeable yellow pristine MAPbI_3_ film, suggesting a partial perovskite degradation to PbI_2_ owing to the action of water and oxygen molecules. The enhanced stability of the MAPbI_3_+4% BHT film under ambient conditions can be attributed to the antioxidant and hydrophobic properties of BHT. The low BDE of the phenol-hydrogen in BHT deactivates the intermediate channels responsible for perovskite degradation, improving the antioxidant properties of perovskite films. Additionally, the hydrophobic functional groups of BHT (tert-butyl in benzene rings and the alkyl groups) improve hydrophobicity^33^. As shown in Fig. S6, the contact angle of the MAPbI_3_+4% BHT film (48.66°) is larger than that of the pristine MAPbI_3_ film (38.83°), suggesting that BHT effectively resists the degradation of perovskite films by water erosion.

### Enhanced ASE performance based on the dual action of BHT and moisture

**Fig. 2:**
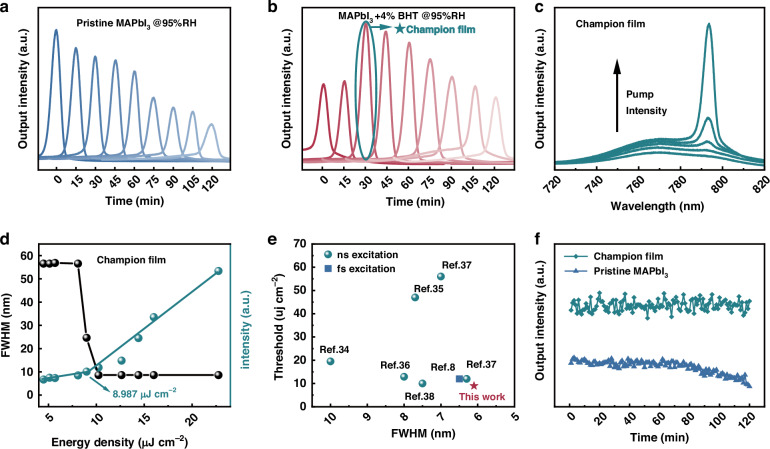
ASE and stability characterizations. Time evolution ASE spectra of **a** pristine MAPbI3 @95% RH and **b** MAPbI3+4% BHT @95%RH in 120 min. **c** Emission spectra under different pump energy and **d** ASE threshold of the champion film. **e** Comparisons of the ASE threshold of the champion film with other reported perovskites. **f** ASE intensities of pristine MAPbI3 and champion films illuminated continuously for 120 min with the pump density of 720 μJ cm⁻^2^

The moisture resistance property of perovskite films can be improved by introducing BHT as an additive for achieving high performance and stable ASE under ambient conditions. Although moisture negatively affects perovskite stability, it can also be used to promote perovskite crystallization. Hydrophobic BHT is expected to increase the interaction time between water and perovskite, slowing the crystallization process of perovskite and improving its morphology, crystallization and film quality. The active role of water molecules in the growth process of perovskite and the corresponding device performance were analyzed by selecting pristine MAPbI_3_ and MAPbI_3_+4% BHT films to study their in situ ASE spectra under humid treatment. Both films maintain stable ASE intensities for 2 h under low relative humidity (RH) conditions (11% and 54% RH, Fig. S7). However, the ASE intensities of the pristine MAPbI_3_ film decease substantially under high humidity (95% RH, Fig. 2a) owing to the degradation of the film by excess water molecules in the absence of BHT. Alternately, the specific ASE intensities of the MAPbI_3_+4% BHT film do not decrease under 95% RH (Fig. 2b), but surprisingly, increase with prolonged time. Especially, a substantial increase in the output intensity can be obtained after 30 min of 95% RH treatment with the ASE intensity 1.7-fold of its initial level, which strongly confirms that the hydrophobicity of BHT significantly enhances the ASE performance of the film, rather than its antioxidant property. The MAPbI_3_+4% BHT film treated under a 95% RH environment for 30 min was considered as the champion film for further study. The ASE performance of the champion film are improved comprehensively compared with that of the pristine MAPbI_3_ film (Figs. 2c, d, and S8). The ASE threshold decreases from 56.88 to 8.987 μJ cm^−2^, gain increases from 37.44 to 103.73 cm^−1^ and loss decreases from 14.51 to 4.36 cm^−1^. The obtained ASE threshold of 8.987 μJ cm^−2^ is the lowest threshold by nanosecond pulse laser excitation under ambient conditions reported to date (Fig. 2e and Table S2)^8,34–38^. Furthermore, as is shown in Fig. S9, the ASE performances of champion film under femtosecond laser excitation are measured, and the ASE threshold is obtained to be 7.11 µJ cm^−^^2^, which can be compared to the majority excellent ASE thresholds reported under similar conditions (Table S3). It is worth noting that nanosecond laser sources with sufficient energy above the ASE and lasing threshold are feasibly obtained at a low cost, which is available to develop continuous optically or electrically pumped lasers. Additionally, as displayed in Figs. 2f, S10 and S11, the champion film under the dual action of BHT and moistrue exhibits good stability, strongly proving the comprehensive effect of the antioxidant and hydrophobic properties of BHT. In this work, the ASE thresholds of the champion film were further tested at continuous excitation for 120 min under the pump density of fivefold initial ASE threshold. As shown in Fig. S12, the ASE threshold is maintained nearly 100% of the initial value at continuous excitation for 60 min, followed with a small increases at 120 min, which confirms the excellent photostability of the champion film. During the ASE performance testing process, light pumping will generate the heat. The long-term stability measurement result under sustained optical excitation indicates the ASE intensity of the champion film remains virtually unchanged throughout the entire test period, as shown in Fig. 2f above, confirming that the heat generated during the test does not affect the ASE performance. Furthermore, as shown in Fig. S13, the ASE threshold of the champion film after being heated on a hot plate at 100 °C in air for 30 min exhibits negligible change, while that increases 58% for the pristine MAPbI_3_ film, which further confirm the excellent thermal-stability of our champion sample.

**Fig. 3:**
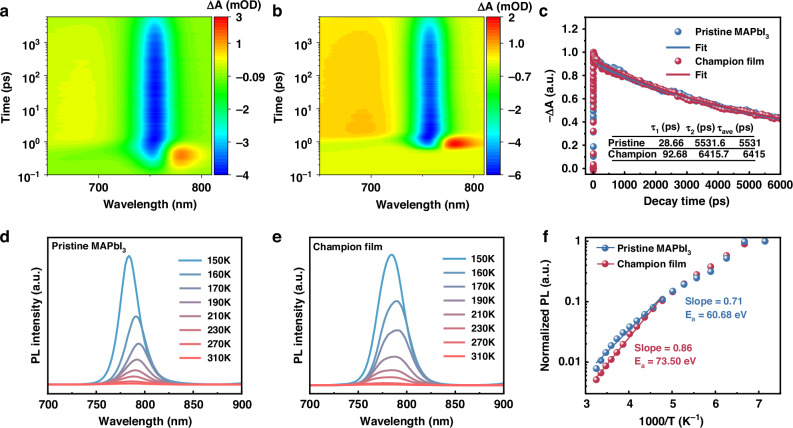
Excitation dynamics analysis. **a**, **b** Representative pseudo colour TA spectra plots, **c** normalized band-edge photobleaching kinetics, **d**, **e** the temperature-dependent PL spectra and **f** Arrhenius plots of temperature-dependent integrated PL intensity of pristine MAPbI3 and champion films

The photo-generated carrier dynamics of the pristine MAPbI_3_ and champion films were analyzed via ultrafast pump-probe transient absorption (TA) spectroscopy (Fig. 3a, b). A major negative absorption changing ground-state bleach (GSB) feature (blue signal) appears at about 760 nm. The GSB peak dynamics are analyzed via the time-evolution TA spectroscopy (Fig. 3c), and bi-exponential fit (${\rm{I}}={{\rm{A}}}_{1}{{\rm{e}}}^{-{\rm{t}}/{{\rm{\tau }}}_{1}}+\,{{\rm{A}}}_{2}{{\rm{e}}}^{-{\rm{t}}/{{\rm{\tau }}}_{2}}$) to the time profile is conducted. The normalized band-edge photo-bleaching kinetics indicate well-fitted dynamics of both films (the fit degree exceeds 99%) and the fitting parameters are shown in Table S4. Two distinct dynamic decay processes are observed: a rapid decay attributed to Auger recombination, which is due to the higher excitation power density, and a slower decay process arising from the combined effects of trap-mediated exciton recombination and bimolecular radiative recombination^39^. The champion film exhibits Auger recombination lifetime (*τ*_1_) and average carrier lifetime (*τ*_ave_) of 92.68 ps and 6145 ps, longer than that of the pristine MAPbI_3_ film (28.66 ps and 5531 ps). As shown in Table S4, the amplitudes for the rapid decay and slower decay are A_1_ and A_2_, respectively, and the proportion of A_2_ for both films exceed 99%, which indicates that the difference of the average lifetime primarily arises from the slower decay component. Furthermore, the raw TA kinetics data (non-normalized) and the detailed bi-exponential fitting parameters are shown in Fig. S14, which shows a similar TA results with the normalized band-edge photobleaching kinetics. For semiconductor lasers, optical gain is significantly limited by non-radiative recombination pathways, including trap states, phonon scattering, and Auger recombination^40^. The extended lifespan of the champion film indicates its higher crystallinity and lower defect density, which significantly suppress Auger recombination and non-radiative recombination induced by carrier accumulation, while markedly enhancing charge carrier transport efficiency and extend carrier lifetime. All of the above results suggest that a weaker non-radiative recombination process within the champion film, which confirms its high crystallinity and low defect density.

The formation and recombination of excitons are key processes in semiconductor materials for achieving laser emission. The energy released during exciton recombination can be converted into photons. This energy is closely related to the threshold and efficiency of a laser. A high binding energy (*E*_*b*_) of excitons enhances the efficiency of the exciton recombination during luminescence. Thus, excitation is achieved at a low carrier concentration, which reduces the threshold power. The effect of the dual action of BHT and moisture on the *E*_*b*_ and intrinsic luminescence mechanism of both films were analyzed via temperature-dependent PL spectroscopy in the temperature range of 150–310 K. Figure 3d, e shows that both films exhibit similar temperature-dependent evolution. PL intensities decay continuously with increasing temperature, which is attributed to the thermally activated depopulation of exciton levels or thermal activation of non-radiative recombination centers^37,41^. Since PL intensities are inversely proportional to nonradiative lifetimes *τ*∝exp(*E*_*a*_/*kT*), the activation energies *E*_*a*_ can therefore be determined from the slope of an Arrhenius plot of the integrated PL intensity as shown in Fig. 3f^34^. The observed temperature-dependence curve conforms semiconductor characteristics^42,43^, and the regime of strong thermal quenching at high temperatures tends towards a straight line, following an exponential quenching ∝ exp (*E*_*a*_/*kT*). It can be obtained that the *E*_*a*_ of the champion film (~73.50 meV) is higher than that of the pristine MAPbI_3_ film (60.68 meV). The exciton thermal dissociation induces the thermal quenching of PL intensity, so the *E*_*a*_ is nearly equal to the *E*_*b*_, and the *E*_*b*_ of the champion film (~73.50 meV) is higher than that of the pristine MAPbI_3_ film (~60.68 meV). The low *E*_*b*_ of solar cells is beneficial for carrier dissociation at room temperature, while laser devices require a high *E*_*b*_ to accelerate the radiative recombination process of carriers and favor emission behavior. The obtained high *E*_b_ of the MAPbI_3_ film under the dual action of BHT and moisture confirms that water molecules boost the perovskite film quality in the presence of BHT, enhancing ASE performance with an ultra-low ASE threshold and high gain.

**Fig. 4:**
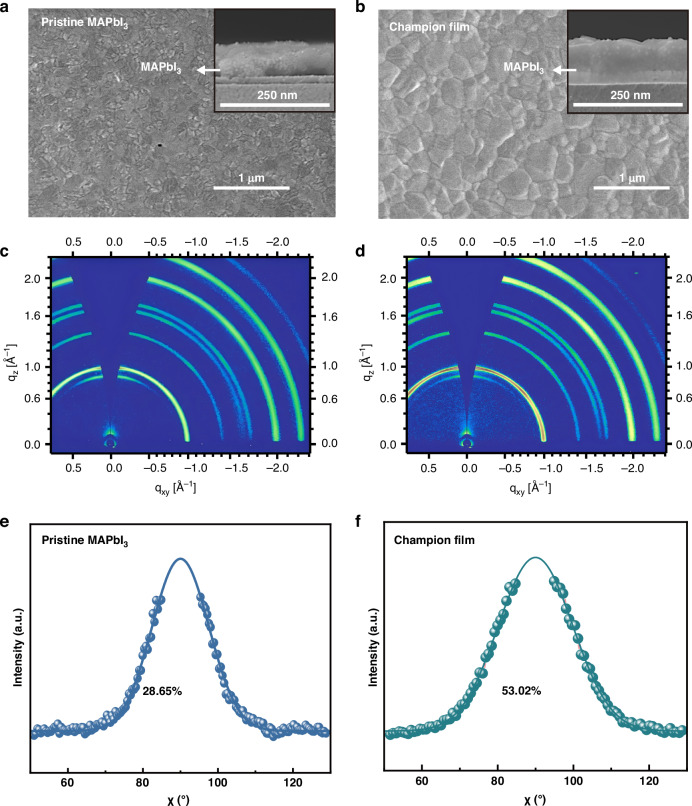
Morphological and crystal orientation characterizations. **a**, **b** Top-view SEM images, **c**, **d** 2D GIWAXS data and **e**, **f** tube integrals at *qxy* ≈ −1Ǻ−1 obtained from the 2D GIWAXS data of the pristine MAPbI3 and champion films. The insets in **a**, **b** are the corresponding cross-sectional SEM images

The morphology of the pristine MAPbI_3_ and champion films analyzed via SEM are presented in Fig. 4a, b, respectively. The champion film exhibits a close-packed distribution of large crystal grains, while the pristine MAPbI_3_ film exhibits uneven grain distribution and some pinholes. Additionally, the cross-sectional SEM images (inset of Fig. 4a, b) of the films demonstrate irregularly arranged small grains in the pristine MAPbI_3_ film while monolithic vertically packed large perovskite grains in the champion film with few grain boundaries. The grain boundary reduction in carrier transport minimizes the scattering loss by disordered states and recombination loss by ionic impurities or traps at grain boundaries, which are beneficial for population inversion in ASE and lasing devices^44,45^. Furthermore, it can be observed that thicknesses for the pristine MAPbI_3_ and champion films are about 143 and 148 nm, respectively; the comparable thicknesses for both films excludes the influence of thickness on the optical properties like PL and ASE. The 2D GIWAXS images (Fig. 4c, d) exhibit distinct diffraction characteristics of crystal phases for both perovskite films. Notably, the *q*-coordinates of the Bragg peaks of both films are consistent, indicating that BHT molecules barely percolate into the lattice, despite their intermolecular interactions in the precursor solution. Whereas, the pristine MAPbI_3_ film exhibits a heterogeneous distribution, while the champion film shows superior orientation. In particular, the (110) plane of the champion film demonstrates a more prominent alignment (perpendicular to the substrate) than that of the pristine MAPbI_3_ film.

In order to reveal the angular information of the (110) plane, tube cuts with a specific angular width (*q*_xy_ ≈ −1Ǻ^−1^) are performed and fitted using Gaussian functions, as shown in Fig. 4e, f. The result reveals that ~53.02% of the crystals in the champion film are perpendicularly orientated, aligning the crystals with the direction of photon movement and minimizing grain boundaries in the photon transmission direction. However, only 28.65% of the crystals exhibit such perpendicular orientation in the pristine MAPbI_3_ film. Thus, more perovskite crystallization sites by the dual action of BHT and moisture facilitate the formation of an optimized perovskite film with enhanced crystallinity, favorable orientation of the crystals and high coverage, which are essential for preparing gain materials with high performance and improving light absorption, exciton lifetime and light propagation.

### Synergistic mechanism of the dual action of BHT and moisture

From the above, the strategy of water molecules and additive dual action improves the quality of perovskite films, reducing the density of defect states and achieving enhanced ASE performance. Specially, the MAPbI_3_+4% BHT perovskite films under high humidity treatment reach their optimum with an ultra-low threshold at nanosecond pulse excitation. It is evident that BHT promotes the active role of water molecules in the film formation process, which increases the coverage uniformly and promotes the crystallization of perovskite crystals, thus forming high-quality perovskite films. Therefore, the synergistic mechanism of the dual effects for BHT and moisture on film quality and ASE are worth further investigation.

**Fig. 5:**
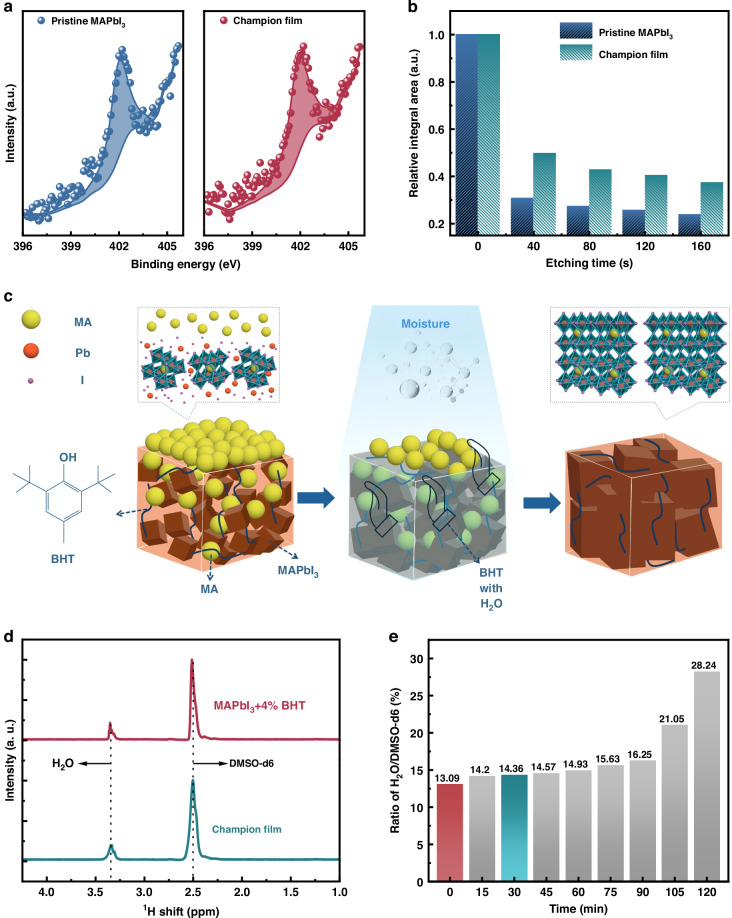
Mechanism exploration. **a** Depth profile of N 1*s* peak in XPS spectra and **b** relative integral area of the MA signal peak in different etching time of pristine MAPbI3 and champion films. **c** Schematic illustration for the crystallization of MAPbI3 under the dual action of BHT and water. **d** 1H NMR spectra of MAPbI3+4% BHT and champion films. **e** Ratio of the relative integral area of H_2_O and DMSO-d6 from NMR results at different moisture-treated time

The influence of water molecules on the spatial distribution of the chemical species in both films was analyzed via depth-dependent XPS (Fig. 5a). The peak representing the C–N bond at 402.14 eV is assigned to MA, and the relative integral area of the signal peak presents the diffusion degree through the moisture-treated perovskite film in the vertical direction of the MA cation^20^. Figure 5b shows the trend of MA content variation with the film depth, where MA gradually decreases with etching depth in both films. However, the champion film presents a stronger MA signal than the pristine MAPbI_3_ film across the etching depth, indicating that moderate water molecules can be in favor of vertical diffusion for organic cation in the film growth. It is safe to assume that water initially interacts with the top organic salt methylammonium iodide (MAI) via hydrogen bonding. And more mobile hydrated salt then rapidly diffuses to the below lead iodide (PbI_2_) molecules, then distributing the crystalline sites of perovskite throughout the film rather than exclusively at the PbI_2_-MAI interface^20^. Moreover, the increased mass transport might be responsible for a rapid precursor supply essential for perovskite growth, resulting in the formation of large perovskite grains (Fig. S1a)^46^. As the schematic illustration shown in Fig. 5c, BHT acts like a cross-linked mesh owing to its hydrophobicity, prolonging the interaction duration between water and perovskite. Therefore, in high-humidity environments, BHT resists the onslaught of waters to perovskite films. In the full crystallization process of MAPbI_3_, the positive effect of water molecules is fully utilized under the motivation of BHT, thus improving the film quality and finally exhibiting ASE enhancement.

The influence of moisture on perovskite crystal formation was further explored by an in situ analysis of the relative amount of absorbed water molecules of the MAPbI_3_+4% BHT film from its initial stage to different moisture-treated stages via ^1^H NMR. In this work, the as-prepared film was dissolved in Dimethyl Sulphoxide-d_6_ (DMSO-d_6_), a solvent for NMR test, and the corresponding NMR spectra were recorded in Fig. 5d. The ratio of the integrated areas of the characteristic peaks of H_2_O to DMSO-d_6_ indicated the amount of water molecules that were absorbed. As shown in Fig. 5e, as the humidity treatment time prolongs, the absorbed water molecules increase at a very slow rate within the first 90 min. Such results indicate that water molecules do gradually enter the perovskite film and contribute to the perovskite crystallization process. On the other hand, it once again confirms that the hydrophobicity of BHT plays an important role in resisting the onslaught of water on the perovskite films, which allows the perovskite film to recrystallize slowly under high humidity atmosphere thereby exhibiting a superior crystal structure. Furthermore, the measurement of H_2_O content in the sample was conducted by Fourier-transform infrared (FTIR) spectra. As shown in Fig. S15, the FTIR signal at about 1650 cm^−1^ represents the bending vibration mode of H_2_O and the signal intensity can be used as an indicator of H_2_O content^47^. It is noted that the intensities for FTIR signals of H_2_O for champion film are weaker than those of the pristine MAPbI_3_ film both before and after the humidity treatment, which once again confirms the hydrophobicity of BHT. The unique role of BHT in controlling water intakes highlights water molecules and additives dual action on the quality improvement of perovskites, leading to much suppressed defect states, which is crucial for inhibiting Auger recombination and achieving state-of-the-art lasing devices with high efficiencies.

## Discussion

In summary, this work demonstrates that the synergistic regulation of moisture and BHT additive enables methylammonium lead iodide (MAPbI₃) films with exceptional optoelectronic properties. Through optimized BHT incorporation (4 wt%) combined with controlled 95% relative humidity treatment, we achieve an unprecedented ASE threshold of 8.987 μJ cm⁻² under nanosecond pulse excitation—representing an 84% reduction compared to pristine films (56.88 μJ cm⁻²) and the lowest value reported to date. The dual-triggered films simultaneously exhibit enhanced net modal gain (103.73 cm⁻¹) and complete preservation of ASE intensity after 30-day ambient storage. Mechanistic investigations reveal that BHT’s hydrophobic character extends water-perovskite interaction kinetics, facilitating vertical methylammonium cation diffusion through the film depth and preferential (110) crystal orientation with 53.02% perpendicular alignment. These structural advantages suppress non-radiative recombination centers, yielding 11% prolonged carrier lifetimes and enhanced exciton binding energy. Our findings transform moisture from a degradation agent into a crystallization promoter, establishing a materials design paradigm for perovskite lasers that concurrently deliver ultralow operational thresholds and robust stability.

## Materials and methods

### Materials

MAI and PbI_2_ were procured from Xi’an Polymer Light Technology Corp and were used without further purification.

### Film fabrication

The quartz substrates were consecutively washed for 15 min using detergent, de-ionized water, ethanol, acetone and isopropanol, dried under a nitrogen flow, and treated using ultraviolet (UV)-ozone for 30 min. Methylammonium acetate (MAAc) was synthesized following the conventional reported method^48^. A mixture of 178.45 mg PbI_2_ and 61.55 mg MAI in 1 mL MAAc was stirred at 60 °C for 24 h to obtain the precursor solution. The MAPbI_3_ film was fabricated via one-step spin coating under ambient conditions as mentioned: MAPbI_3_ precursor solution (60 µL with the concentration of 240 mg mL^−1^) was spin coated on a quartz substrate under 4000 rpm for 20 s at 70 °C. The substrate was annealed at 100 °C for 5 min to form the MAPbI_3_ film. The MAPbI_3_+x% BHT films were prepared using the above-mentioned process but by adding different amounts of BHT to the precursor solution. The MAPbI_3_+4% BHT film was prepared by adding 9.6 mg BHT to the precursor solution. The samples were exposed to specific RH conditions for moisture treatment. Sealing the atmosphere with a saturated salt solution to create different humidity environments. The specific RH conditions were 11% RH with lithium chloride (LiCl), 54% RH with magnesium nitrate (Mg(NO_3_)_2_), 75% RH with sodium nitrate (NaNO_3_) and 95% RH with potassium nitrate (KNO_3_).

### Characterization of perovskite films

The XRD spectra of the samples were recorded using a Rigaku D/MAX 2500 PC X-ray diffraction instrument, Cu Kα radiation with a wavelength of 0.154 nm. The surface morphology of the samples was analyzed using a Hitachi Regulus 8220 scanning electron microscope. The AFM results of the samples were recorded using a Bruker Dimension EDGE. UV-vis spectroscopy was performed using a Hitachi U-4100 spectrophotometer. Steady-state PL spectroscopy was performed using a LS-50B luminescence spectrometer manufactured by PerkinElmer. GIWAXS was performed using a XEUSS SAXS/WAXS system from Xenocs, France. A Ti:sapphire femtosecond amplifier (800 nm center wavelength, 35 fs pulse width and 1 kHz repetition rate) and an optical parametric amplifier were used as the laser light sources for the commercial ultrafast transient absorption equipment (Helios Fire, Ultrafast System).

### ASE tests

A solid-state Nd:YAG laser (minite II Q-switched Nd:YAG) @ 355 nm delivering 3–7 ns pulses with a repetition rate of 10 Hz was used. The pump intensity was measured by a calibrated laser power and energy meter (Gentec). A cylindrical lens was used to focus on the light beam to shape a narrow stripe. The emitted light was corrected through an optical fiber and directed into an imaging spectroradiometer (Horiba, iHR-320). The gains of the sample were measured via variable stripe length methods at room temperature in ambient conditions. The length of the excitation streak was varied through an adjustable slit driven by a micrometer at the focal line of the cylindrical lens.

## Supplementary information

Supplementary Information

## Data Availability

Data available with the paper or supplementary information.
