# Supplementary material for: Break-through amplified spontaneous emission with ultra-low threshold in perovskite via synergetic moisture and BHT dual strategies
Source: Light Sci Appl. 2026 Feb 2;15:99. doi: 10.1038/s41377-025-02171-8 (PMC12862052; doi:10.1038/s41377-025-02171-8)
Supplement: Supplementary file 1 — Supplementary Information [file 41377_2025_2171_MOESM1_ESM.docx]

**Supplementary Information for**

**Break-through Amplified Spontaneous Emission with Ultra-low Threshold in Perovskite via Synergetic Moisture and BHT Dual Strategies**

*Dingke Zhang^1*^, Rui Li^1^, Haoyue Luo^1^, Zhen Meng^1^, Jingwen Yao^1^, Hongfang Liu^2^, Yexiong Huang^1^, Shuaiqi Li^1^, Peng Yu^1^, Jie Yang^1^, Mingyu Pi^1^, Shencheng Fu^2,3*^, Zhenxiang Cheng^4^, Yichun Liu^2,3^*

^1^School of Physics and Electronic Engineering, Chongqing Normal University, Chongqing, 401331, China

^2^School of Physics, Northeast Normal University, Changchun, 130024, China

^3^State Key Laboratory of Integrated Optoelectronics, Northeast Normal University, Changchun, 130024, China

^4^Institute for Superconducting and Electronic Materials Faculty of Engineering and Information Sciences University of Wollongong Innovation Campus, Squires Way, North Wollongong, NSW 2500, Australia

* Corresponding author.

E-mail: zhangdk@cqnu.edu.cn, fusc515@nenu.edu.cn


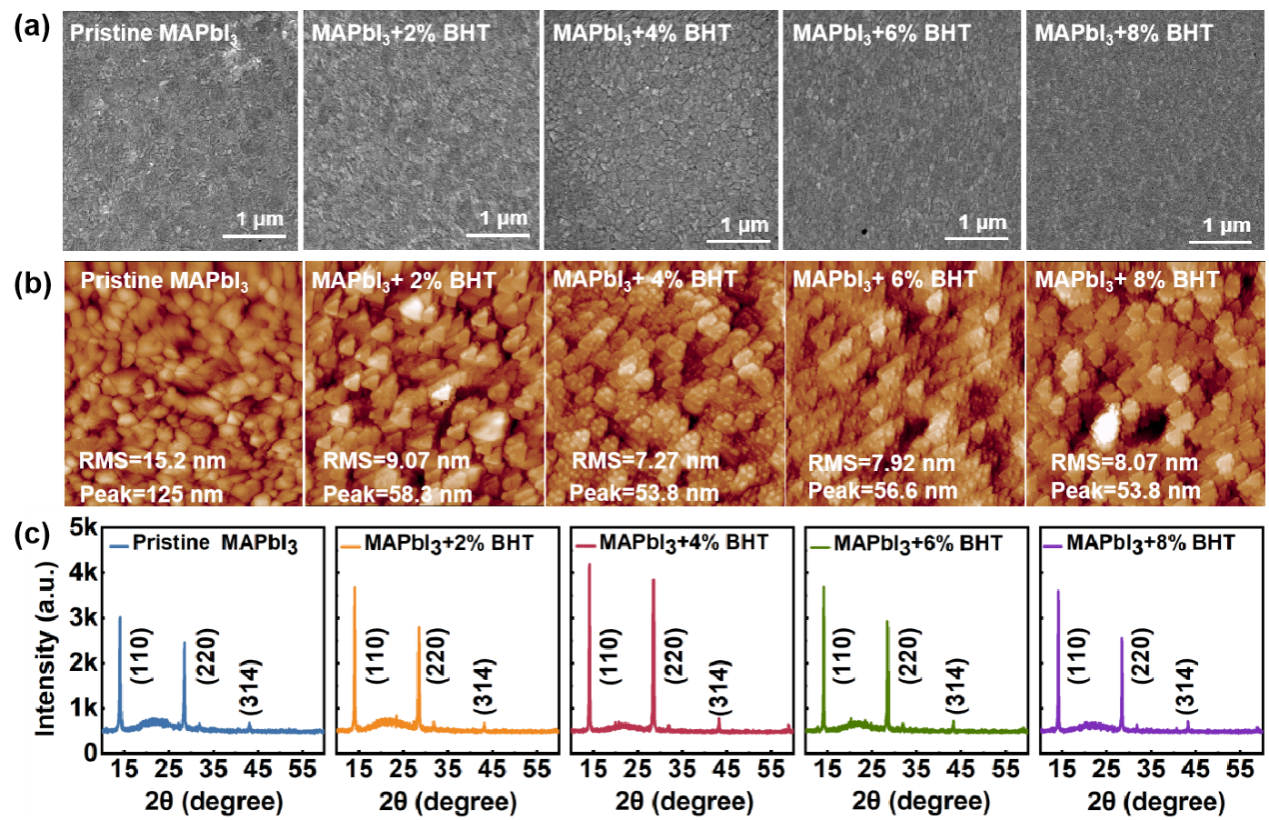


**Fig. S1** (a) SEM images, (b) AFM images and (c) XRD results of the MAPbI_3_ perovskite films with varying BHT concentrations (0, 2, 4, 6, 8 wt%).


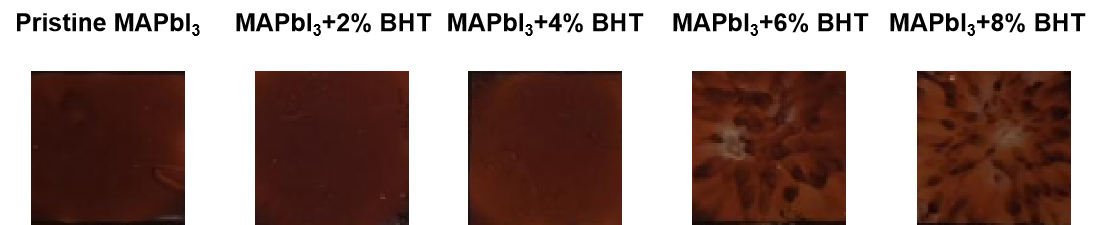


**Fig. S2** Photos of MAPbI_3_ perovskite films with different BHT concentrations.


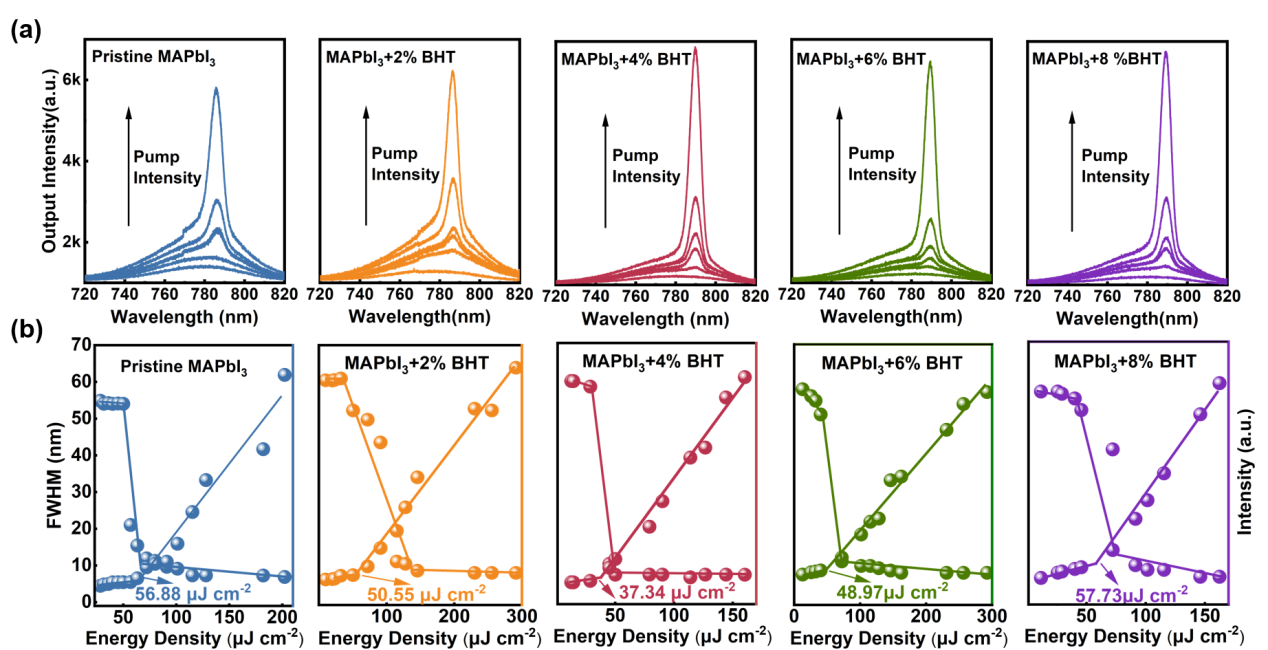


**Fig. S3** (a) Emission spectra under different pump energies and (b) The ASE threshold and FWHM behaviors of the of MAPbI_3_ films with different BHT concentrations.

The ASE measurement details are as follows: The nanosecond pulse laser was delivered by a solidstate Nd:YAG laser (minite II Q-switched Nd:YAG) @355 nm delivering 3-7 ns pulses with a repetition rate of 10 Hz. The output pulse energy of the pumping laser was controlled using neutral density filters. The pump intensity was measured by a calibrated laser power and energy meter. A cylindrical lens was used to focus on the light beam to shape a narrow stripe. The excitation beam was focus on the sample vertically. The emitted light was corrected through an optical fiber and directed into an imaging spectroradiometer (Horiba, iHR-320).


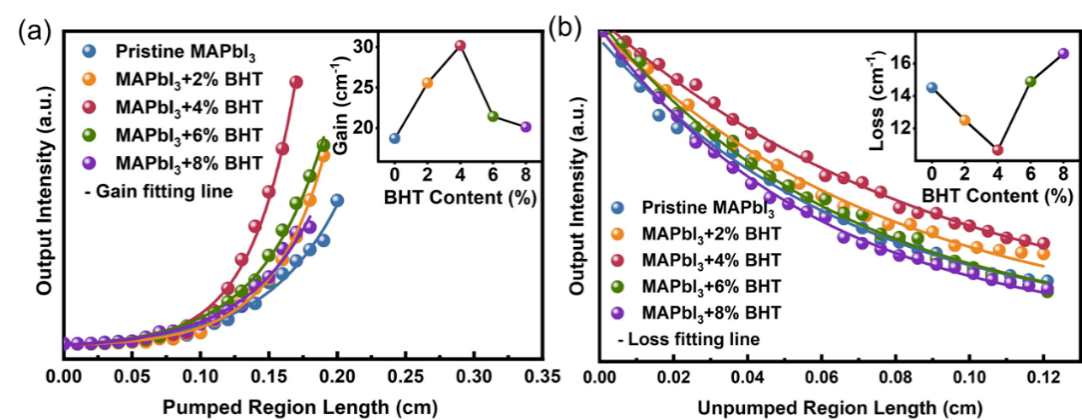


**Fig. S4** The gain and loss of the MAPbI_3_ films with different BHT concentrations.

Both the optical gain and loss coefficients were measured via variable stripe length method under consistent excitation conditions using a nanosecond pump laser (355 nm, with a pump stripe measuring 0.8 mm in width and 5 mm in length, pump fluence of 720 μJ cm^-2^.) The deliberate choice of pump fluence was made to ensure a sufficiently strong ASE signal with a high signal-to-noise ratio, allowing us to clearly resolve the ASE emission peak and accurately extract the gain/loss coefficients. In both measurements, the emission intensity was recorded at a fixed wavelength corresponding to the ASE peak rather than integrating the entire emission spectrum. This method ensures accurate comparison between samples and prevents confusion with spectrally varying features. The test details are as follows:^S1^

The net gain of film was determined by measuring the ASE intensity pumped by laser with variable stripe length. The relationship between emission intensities and pumped length is mathematically represented by the equation:

$I=\frac{A\left( \lambda\right)I_{P}}{G\left( \lambda\right)}(e^{G\left( \lambda\right)L}-1)$ Equation (S1)$s_{1}=-\frac{\upsilon}{E},$

where *A(λ)* is a constant linked to the spontaneous emission cross-section, *I_P_* is the ASE intensity, *G(λ)* is net gain coefficient, and *L* is the length of the pumped stripe. Besides, the optical losses were determined by tracking the variation in emission intensity along the excitation region as it moves from the edge of the film. This measurement relies on the principle that emission remains consistent at the end of the pump stripe, allowing the observed signal at the film's edge to adhere to the Beer-Lambert law, expressed as equation:

$I = I_{0} e^{-\alpha x}$ Equation (S2)

Here, *α* is the waveguide loss coefficient, and *x* is the unpumped region length of distance from the end of the pump area to the sample's edge.

**Table S1**. The values of gain and loss of the MAPbI_3_ films with different BHT concentrations.

| Sample | Pristine MAPbI_3_ | MAPbI_3_+2% BHT | MAPbI_3_+4% BHT | MAPbI_3_+6% BHT | MAPbI_3_+8% BHT |
| --- | --- | --- | --- | --- | --- |
| Gain  (cm^-1^) | 18.71 | 25.58 | 30.18 | 21.42 | 20.13 |
| Loss  (cm^-1^) | 14.51 | 12.49 | 10.66 | 14.88 | 16.60 |


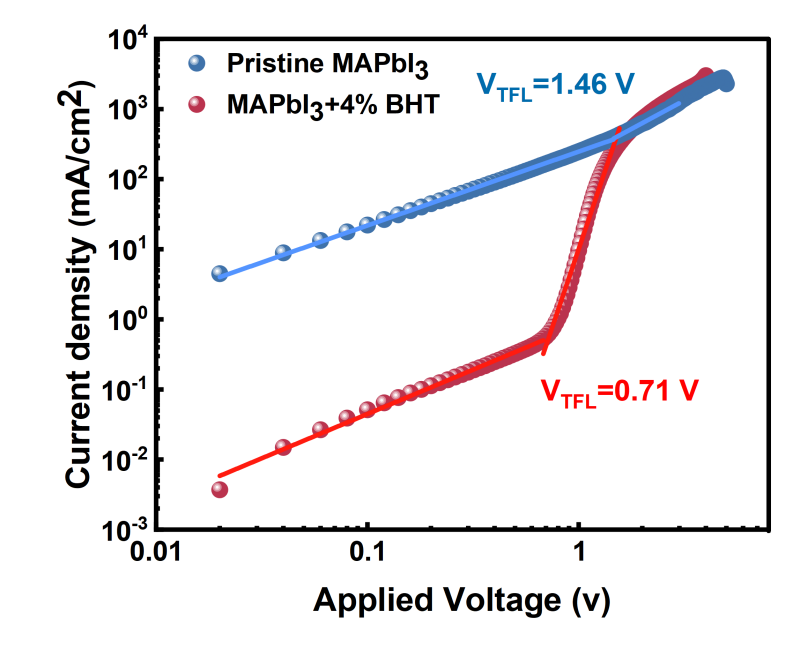


Fig. **S5** Space charge limited current measurements for the pristine MAPbI_3_ and MAPbI_3_+4% BHT perovskite films.


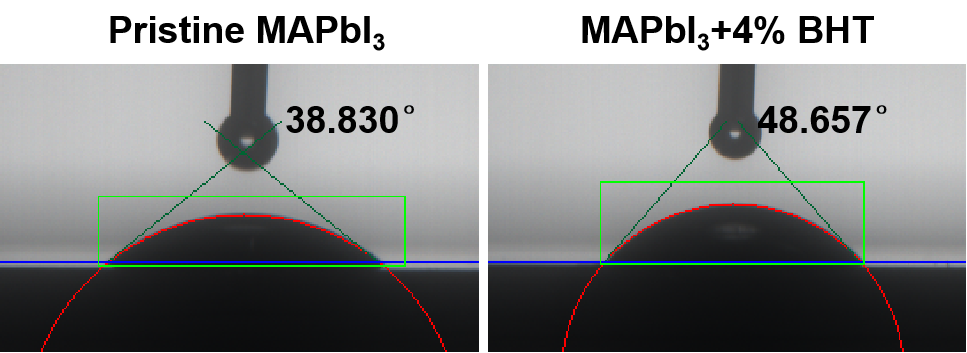


**Fig. S6** The contact angles for the pristine MAPbI_3_ and MAPbI_3_+4%BHT films.


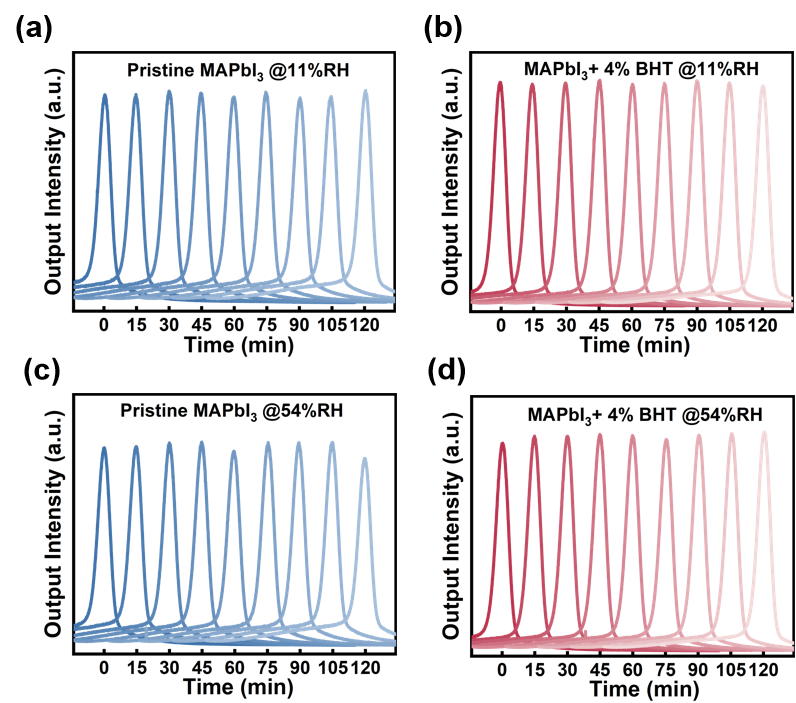


**Fig. S7** ASE spectra from the pristine MAPbI_3_ and MAPbI_3_+4%BHT films treated at (a and b) 11% RH and (c and d) 54% RH for 2 h.


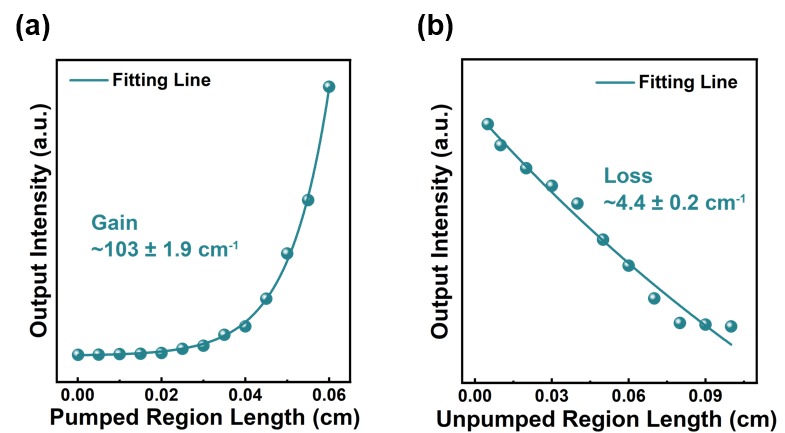


**Fig. S8** (a) gain and (b) loss of the champion film.

**Table S2**. Comparisons of ASE thresholds for the champion film in this work with other reported MAPbI_3_ samples.

| Sample | ASE threshold  (μJ cm⁻^2^) | Laser style  (ns or fs) | Reference |
| --- | --- | --- | --- |
| MAPbI_3_-MAAc | 56 | ns | S1 |
| MAPbI_3_-MAP | 12 | ns |  |
| MAPbI_3_ | 55.9 | ns | S2 |
| PMMA/MAPbI_3_ | 54.4 | ns |  |
| MAPbI_3_/PMMA | 34.4 | ns |  |
| PMMA/MAPbI_3_/PMMA | 12.9 | ns |  |
| MAPbI_3_ | 51 | ns | S3 |
| MAPbI_3_ | 12±2 | fs | S4 |
| MAPbI_3_ | 6 | fs | S5 |
| MAPbI_3_ | 56.88 | ns | This work |
| MAPbI_3_+4%BHT | 37.34 | ns |  |
| Champion films | 8.987 | ns |  |


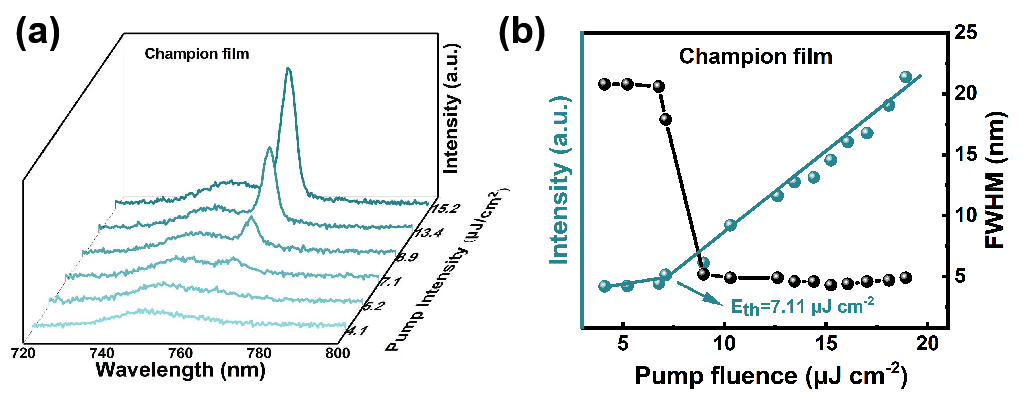


**Fig. S9** (a) Emission spectra under different pump energies and (b) The ASE threshold and FWHM behaviors of the of champion film under femtosecond laser excitation.

**Table S3**. Comparisons of ASE thresholds under femtosecond laser excitation for the champion film in this work with other reported perovskite films.

| Sample | ASE threshold (μJ cm⁻^2^) | Reference |
| --- | --- | --- |
| MAPbI_3_ | 12±2 | S4 |
| MAPbI_3_ | 6 | S5 |
| CsPbBr₃ | 31.5 | S6 |
| CsPbBr₃-PABr | 25.45 |  |
| (FAPbI_3_)_0.992_(MAPbBr_3_)_0.008_  (200 nm) | 49.26±0.05 | S7 |
| (FAPbI_3_)_0.992_(MAPbBr_3_)_0.008_  (60 nm) | 5.84±0.05 |  |
| (FAPbI_3_)_0.85_(MAPbBr_3_)_0.15_ | 12.8 | S8 |
| (FAPbI_3_)_0.85_(MAPbBr_3_)_0.15_  +APCl | 5.3 |  |
| Champion film | 7.11 | This work |


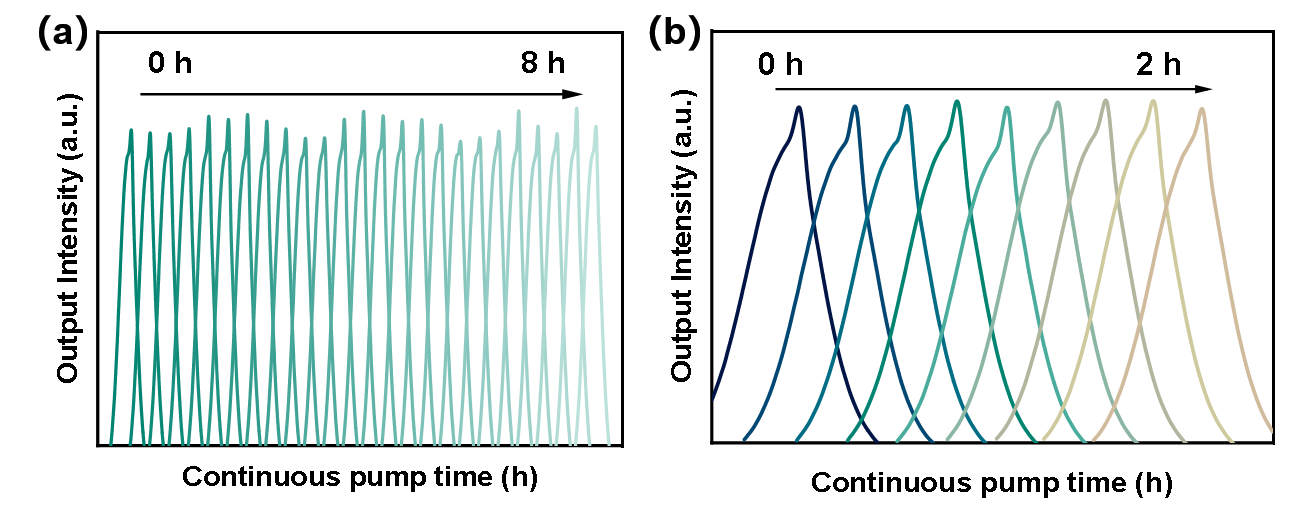


**Fig. S10** (a) Time-depended ASE intensities of the champion film under pumping near threshold (1.11 times the threshold) under continuous excitation in air for 10 h. (b) Time-depended ASE intensities of the champion film under pumping near threshold (1.11 times the threshold) under continuous excitation in air for 2 h at the second day.


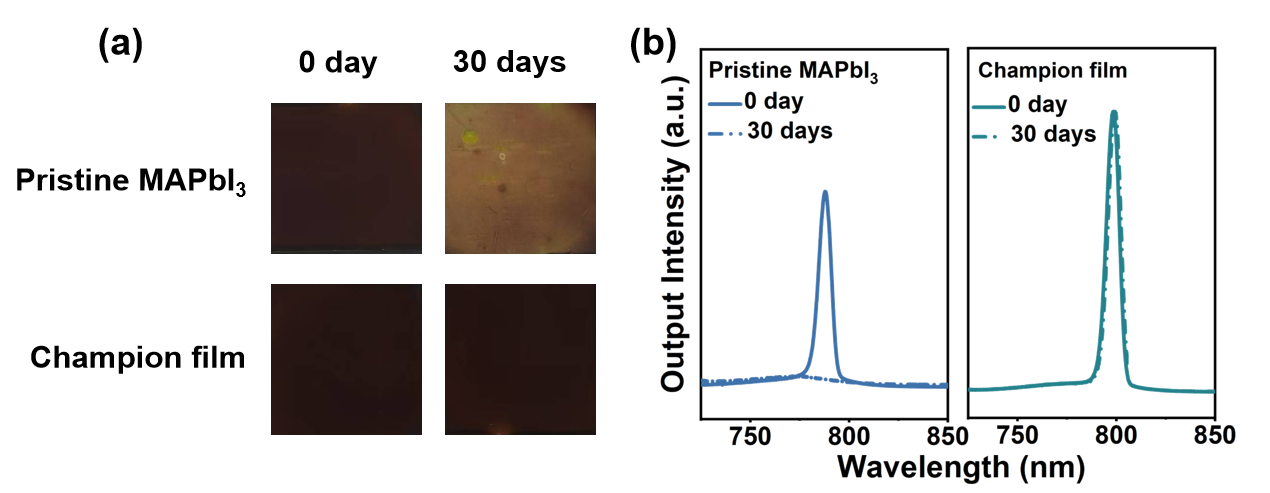


**Fig. S11** (a) Photos and (b) ASE spectra of the pristine MAPbI_3_ and champion films under normal ambient conditions before and after 30 days.


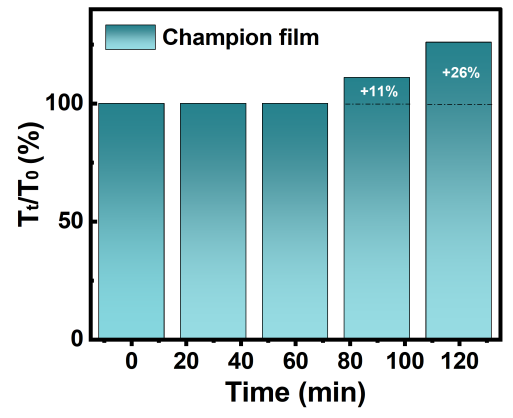


**Fig. S12** Ratio of ASE threshold at continuous excitation for t min (T_t_) and initial value (T_0_) for for the champion film under the pump density of 5-fold initial ASE threshold.


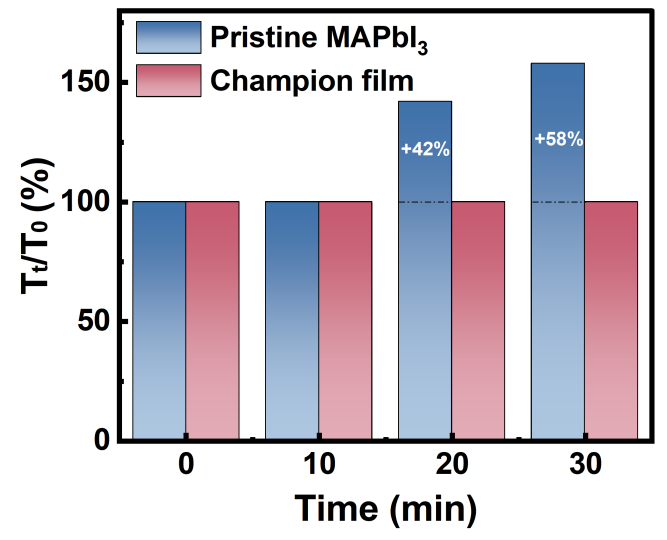


**Fig.** **S13** Ratio of ASE threshold after thermal treatment for t min (T_t_) and initial value (T_0_) before heated at 100 °C in air for 30 min for the pristine MAPbI_3_ and champion films.

**Table S4**. The fitting parameters of TA spectroscopy for GSB peak via bi-exponential fit for the pristine MAPbI_3_ and champion films.

| Sample | τ_1_ (ps) | τ_2_ (ps) | A_1_ | A_2_ | $\frac{A_{1}}{A_{1}+A_{2}}$ | $\frac{A_{2}}{A_{1}+A_{2}}$ | τ_ave_ (ps) |
| --- | --- | --- | --- | --- | --- | --- | --- |
| Pristine MAPbI_3_ | 28.66 | 5531.6 | 2.781E-5 | 0.708 | ＜1% | ＞99% | 5531 |
| Champion film | 92.68 | 6415.7 | 0.005 | 0.759 | ＜1% | ＞99% | 6415 |


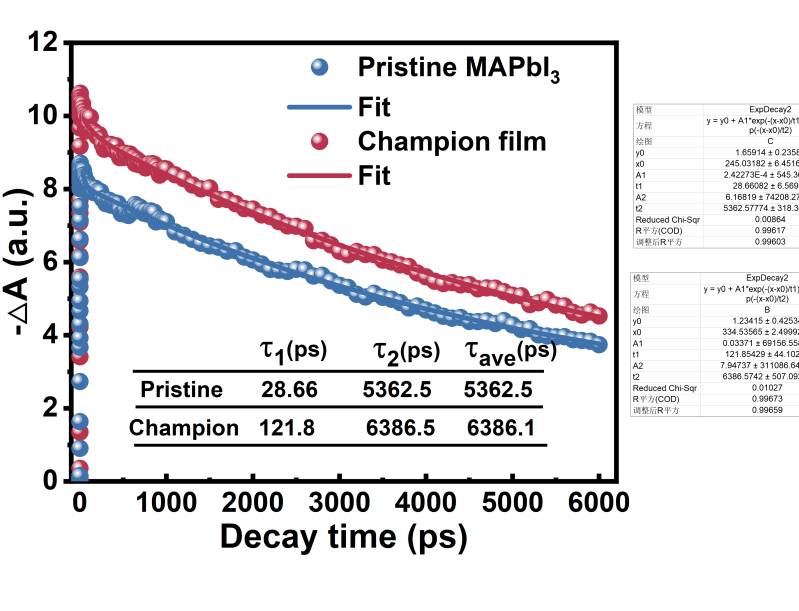


**Fig. S14** Non-normalized band-edge photobleaching kinetics of pristine MAPbI_3_ and champion films.


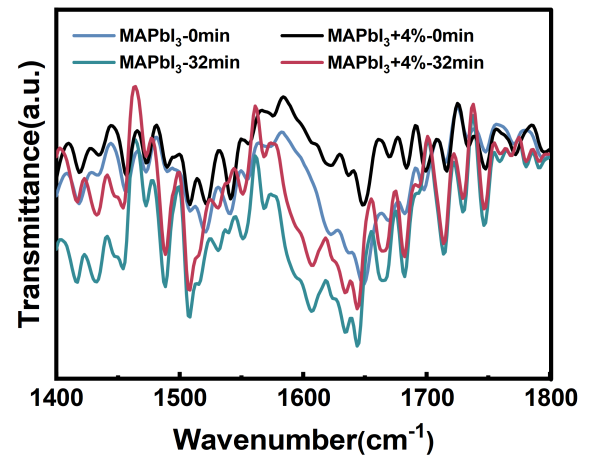


**Fig. S15** FTIR spectroscopy for the pristine MAPbI_3_ and champion films before and after humidity treatment.

**Reference**

[S1] Luo, H., et al. Solution chemistry strategies to construct a stable MAPbI_3_ film toward high performance of amplified spontaneous emission. Chem. Eng. J. 482, 148838 (2024).

[S2] Zeng, X., et al. Achieving low threshold and high optical gain amplified spontaneous emission in MAPbI_3_ perovskite films via symmetric waveguide effect. Adv. Opt. Mater. 10, 2201328 (2022).

[S3] Goldberg, I., et al. Multimode lasing in all-solution-processed UV-nanoimprinted distributed feedback MAPbI_3_ perovskite waveguides. ACS photonics 10, 1591-1600 (2023).

[S4] Feng, N., et al. Light‐Emitting Device Based on Amplified Spontaneous Emission. Laser & Photonics Rev. 17, 2200908 (2023).

[S5] Yuan, F., et al. High stability and ultralow threshold amplified spontaneous emission from formamidinium lead halide perovskite films. ‌J. Phys. Chem. C 121, 15318-15325 (2017).

[S6] Zhang, Y., et al. Influence of the surface modification on carrier kinetics and ASE of evaporated perovskite film. IEEE Photonics Technol. Lett. 35, 285-288 (2023).

[S7] Mao, Y., et al. Enhancing Amplified Spontaneous Emission in Perovskite Thin Films by Thickness Regulation: Insights into Carrier Distribution and Optical Confinement. Adv. Opt. Mater. 2500307 (2025).

[S8] Huang, S., et al. Enhanced Amplified Spontaneous Emission from Perovskite Films by a Multifunctional 4′‐Aminoacetophenone Hydrochloride Additive Assistant Engineering. Adv. Opt. Mater. 12, 2302513 (2024).
